# A Wideband High-Isolation Microstrip MIMO Circularly-Polarized Antenna Based on Parasitic Elements

**DOI:** 10.3390/ma16010103

**Published:** 2022-12-22

**Authors:** Ijaz Khan, Kuang Zhang, Qun Wu, Inam Ullah, Luqman Ali, Habib Ullah, Saeed Ur Rahman

**Affiliations:** 1 School of Electronics and Information Engineering, Harbin Institute of Technology, Harbin 150001, China; 2 BK21 Chungbuk Information Technology Education and Research Center, Chungbuk National University, Cheongju-si 28644, Republic of Korea; 3 College of Electronics and Information Engineering, Nanjing University of Aeronautics and Astronautics, Nanjing 210016, China; 4 School of Electronic Engineering, Xidian University, Xi’an 710071, China

**Keywords:** circular polarization, MIMO, mutual coupling, axial ratio, ECC, WLAN

## Abstract

This work presents a wideband, all-side square-cut square patch multiple-input, multiple-output circularly-polarized (MIMO-CP) high-isolation antenna. The MIMO-CP antenna contains a two-port square cut on all corners of the square patch, and parasitic elements of 9 × 5 periodic square metallic plates are designed and operated. The outer dimensions of the antenna are 40 × 70 mm^2^, and the FR4 substrate height is 1.6 mm. The proposed antenna with the parasitic elements improves impedance matching and enhances S-parameters and axial ratio (AR). In the suggested MIMO-CP antenna, a parasitic element is designed and placed around the antenna periodically to reduce mutual coupling (MC) and improve CP. Simulated results show that the suggested antenna has a wide bandwidth (BW) from 4.89 to 6.85 GHz for S_11_ and was < −10 dB with AR ≤ 3 dB from 5.42 to 6.58 GHz, with a peak gain of 6.6 dB. The suggested antennas have more than 30 dB isolation and a low profile, are affordable, easily made, and are CP. To make a comparison with the measured and simulated results, a MIMO-CP antenna structure was fabricated and tested. The suggested antenna is better in terms of efficiency, envelope correlation coefficient (ECC), diversity gain (DG), channel capacity loss (CCL), and total active reflection coefficient (TARC). The proposed antenna is adequate for WLAN applications.

## 1. Introduction

Nowadays, wireless local area networks (WLAN) are an essential technology for low power consumption, high transmission rates, high signal-to-noise ratios, and high security [1]. Modern wireless communication systems are heavily reliant on multiple-input, multiple-output (MIMO) technology in order to increase channel capacity and meet the growing demand for high-speed, wideband communications [2]. This system is becoming increasingly popular in modern research movements due to its potential benefits, such as high system throughput, enhanced communication reliability, and wideband coverage [3]. The transmitted signal in a wireless communication environment experiences small-scale or large-scale multipath fading, caused by mountains or large structures [4]. However, to reduce the fading effect, MIMO technology revolutionizes this situation by increasing the data rate while maintaining high quality [5,6]. Due to the mutual coupling (MC) effect among the two radiators, it is extremely hard to maintain multiple antennas in a small and dense space when using a MIMO antenna at the user end [7]. MC is caused by the communication of radiation from widely spaced antennas with surface currents flowing on the ground plane. Therefore, it is vital to decrease the MC of the MIMO antenna [8]. This degrades the MIMO antenna diversity performance and increases MC among nearby antennas by causing field correlation and a rise in MC [9].

Several effective ways and methods for MC and increasing the compactness of antenna elements in MIMO antenna systems have been investigated. Several decoupling technologies, such as an F-shaped stub [10], neutralization line (NL) [11], complementary split ring resonator (SPR) [12], defected ground structure (DGS) [13,14], slot [15,16,17], and electromagnetic bandgap structures (EBG) [18,19] have been presented. Recently, metamaterials and metasurfaces [20,21,22] have also been applied to enhanced isolation. In several studies, a dual-band or multi-band MIMO antenna with low MC, a smaller size, and a low ECC has been designed. For WLAN applications, a new MTM-based superstrate design for low MC and better MIMO systems is being studied [23]. The inter-port MC among the antenna elements is improved in [24] via the DGS structure, which offers low MC from −12 to −25 dB at 5.65 GHz. To attain high gain, a smaller size, and better isolation, an artificial magnetic conductor (AMC) is used beneath the V-shaped antenna [25]. In [26], an isolation enhancement of 26 dB was attained by suppressing the MC between two antennas by a unique mushroom-shaped EBG structure. Unluckily, the 0.6λ center-to-center spacing resulted in a large lateral size. Reference [27] designed a compact four-port MIMO antenna for high-isolation and ultra-wideband and introduced T-shaped metamaterial, which enhanced bandwidth and isolation. In [28], a small, closely coplanar waveguide (CPW) MIMO antenna with a comb-shaped MC design also showed a high port isolation of >20 dB. To introduce the decoupling method, a neutralization line is suggested to reduce the MC of the ultra-wideband (UWB) MIMO antenna [29]. To enhance isolation among the patches of MIMO antennas, a mushroom EBG and a fractal-shaped EBG have been studied [30]. However, the entire above-cited antenna’s MC has only presented linear polarization (LP).

Currently, antenna designers are gradually developing and focusing on CP radiators in MIMO systems. The CP antenna demand is growing due to various factors, such as multipath effects, polarization mismatch, and phasing issues. For the ability to maintain a stable connection among the receiving systems and transmitting regardless of the location, CP antennas are frequently preferred in wireless communication over LP antennas [31]. A number of printed antenna shapes using various techniques have been proposed in recent years in response to the rising demand for small antennas with wide BW and ARBW. Because of their simple design, compact size, low profile, uncomplicated design and fabrication, and low cost, as well as their ability to produce CP waves while maintaining a compact antenna size and bidirectional radiation patterns (RHCP and LHCP), printed antennas are gaining popularity for use in indoor environments [32].

Using a MIMO configuration with polarization diversity, the issue of mutual coupling can be resolved more successfully. Nevertheless, the literature has suggested many methods for a MIMO antenna with circular polarization features. In [33,34,35,36,37,38,39,40,41,42,43,44], the authors suggested a MIMO-CP antenna with a high-isolation of more than 10 dB. For WLAN applications of MIMO systems in tiny wearable devices, ground radiation with CP performance has been proposed using a tunable metal strip [33]. MC and CP are attained by modifying the microstrip stubs and annular ring patches of the antennas [34]. In [35], a novel mu-negative metamaterial (MTM) filter-constructed MC technique was used for MIMO CP antennas. Decent isolation among the antennas can be attained with the obtainable decoupling network MTM structure by maintaining compactness, but this has a narrow bandwidth and axial ratio. Moreover, various DGS, slots, and stubs have been used to increase antenna isolation and axial ratio for WLAN application in Endfire and T-shaped slot CP antenna [36] using mirrored F-shaped DGS, three grounded stubs [37], and two optimized 90° apart rectangular slots [38]. The presented antennas in [35] provide low isolation, narrow bandwidth, axial ratio, and low peak gain, and those in [37,38] give narrow bandwidth and low isolation but have disadvantages because of the antenna fabrication complexity and large size. Apart from microstrip patch antenna, numerous types of dielectric resonator (DR) MIMO CP antenna have been referenced in the literature, in which the CP radiation is realized by using a modified circular designed aperture [39], L-shaped DR [40], two rectangular DRs, a single square DR [41], and an F-shaped slot [42]. In conclusion, most of the unidirectional beam MIMO-CP antennas mentioned in the literature have limited operating BW and axial ratio, while those in [42] have wide BW but large sizes. In [43], the MC of a MIMO-CP antenna is considered using parasitic components by placing a parasitic line patch and circular ring parasites in a hybrid decoupling structure [44] for MC reduction and AR.

This article presents a corner square cut of all sides on a square radiator wideband MIMO-CP antenna, which is studied for the WLAN band. The main novelty of the proposed work is that the MIMO-CP antenna is composed of two radiated square corner cut patches and square parasitic elements. The antenna is compact, has a larger 3 dB AR, and has a wider bandwidth. The structure achieved CP by optimizing square-cut corners at the square patch antenna. The square parasitic elements are periodically placed around the two patches for reduced MC among the two MIMO elements and are given a wide AR. The MIMO-CP antenna has a wide bandwidth and axial ratio, and good isolation over the whole frequency range from 4.89 to 6.85 GHz. The proposed antenna is designed for wide bandwidth, low MC, enhanced gain, and necessary impedance bandwidth. Within the frequency range, the results of measurement on the fabricated prototype are near to the simulated result.

## 2. Single Antenna Configuration and Analysis

**Figure 1:**
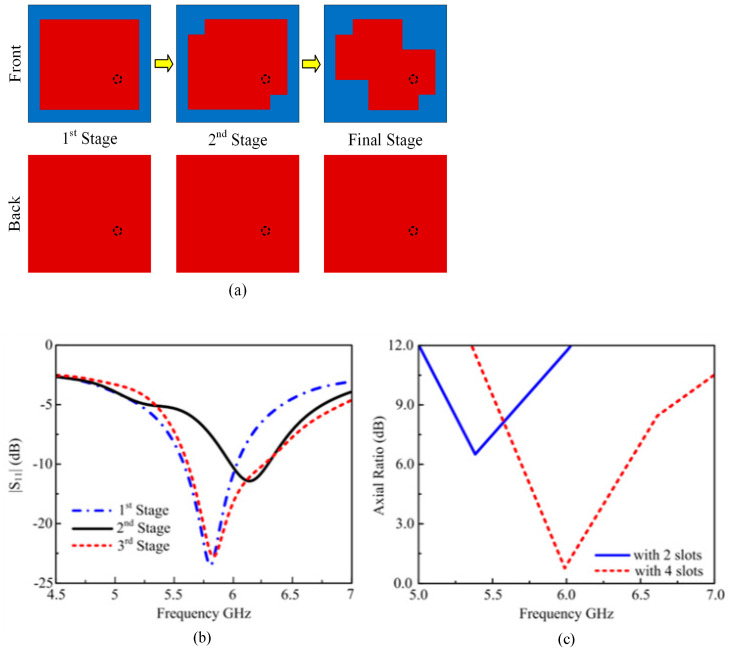
(**a**) Single antenna design evaluation. (**b**) S_11_ results of different single antenna steps. (**c**) single antenna with a cut axial ratio.

In this section, first, a single-CP antenna is designed for WLAN application. The geometry of the single-CP antenna is shown in Figure 1a. The antenna is made up of all corners square cut at the square patch, a ground plane, and an FR-4 substrate; εr = 4.3 and tanδ = 0.025. The thickness of the substrate is 1.6 mm, with outer dimensions of 20 × 25 mm^2^. The antenna is designed with full ground, having dimensions the same as the FR-4 substrate. The antenna ground is attached to the back side of the FR-4 substrate. Initially, the antennas have LP radiation; one of the easy conventional techniques is to apply squares to the cuts at all corners of the square patch to obtain CP.

A wideband antenna has been designed in this paper after different stages for WALN application. Initially, a simple square patch was designed for the WLAN band, which has a narrow band and linear polarization. In the next stage, a two-square cut was inserted into the square patch antenna, with a length and width, W_i_, to obtain wide bandwidth and circular polarization. The antenna after the second stage, resonated at 5.8 GHz and still had no circular polarization. In the third stage, two more square cuts were added to the square patch antenna, which improved the return loss and bandwidth and attained circular polarization. The square-cut corners of the square patch were tuned by G and L_g_ to realize CP performance. The proposed antenna is fed by a 50 Ω coaxial cable via an SMA connector. The outer conductor of the coaxial connector is extended up to the ground, while the inner conductor passes through the dielectric and is soldered to the radiating patch. Figure 1a depicts the initial placement of the feed in the patch’s center and the subsequent adjustment of the x-axis and y-axis by 5 mm and 1.5 mm, respectively, to maximize impedance matching. Figure 1b shows that the single-patch antenna simulated |S_11_| reflection coefficient is below −10 dB impedance BW from 5.45 to 6.3 GHz. Figure 1c shows that the single antenna has been given a 3-dB AR BW from 5.9 to 6.1 GHz. It can be seen that the single-patch antenna has a narrow axial ratio.

## 3. Proposed MIMO-CP Antenna Configuration and Analysis

**Figure 2:**
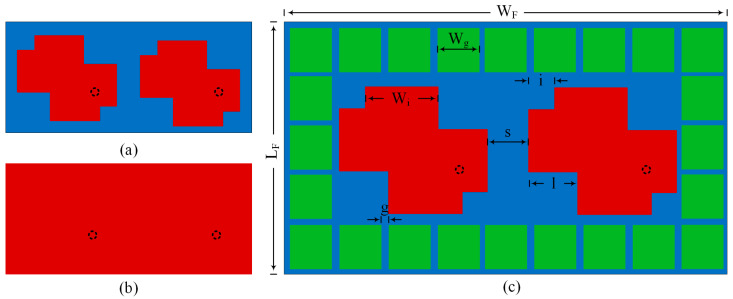
MIMO antenna without parasitic elements: (**a**) top-view; (**b**) bottom-view; (**c**) MIMO antenna with parasitic elements.

The suggested wideband MIMO-CP antenna geometry, design process, and characterization are shown in Figure 2. The dielectric substrate of the antenna has a ground plane along the lower edge and microstrip square patches along the upper edge. By adjusting the design parameters, an optimized single-patch antenna is first modeled, and after that, its characteristics are examined using a MIMO-CP antenna. The radiating elements are two square elements with a corner square cut on all sides of the square patches. 

The progress of the two radiating patches of the MIMO-CP antenna without parasitic elements is shown in Figure 2. Depending on the antenna design and how the square cuts are arranged on the square patches, either left-hand CP (LHCP) or right-hand CP (RHCP) will be produced. The MIMO antenna with parasitic elements is shown in Figure 2c. Figure 2b shows the back view of the proposed parasitic elements antenna with the full ground and the same dimensions as the substrate. The antenna dimensions of the proposed antenna are increased due to parasitic elements, which are L_F_ × W_F_ = 40 × 70 mm^2^. The antenna is fed at Ports 1 and 2 using two 50 Ω SMA connectors. The antenna consists of two square patches in which the corners have square cuts in different dimensions to have CP and square parasitic elements periodically arranged around the radiating patch. The area of the square patch is (p × p). The square cut length and width at the square patches are (l × l) and (I × i). The square parasitic elements have periodicity, w_g_, and the distance between the nearby parasitic elements is g and is placed periodically around the patches for improvement. The final dimension of the suggested geometry is shown in Table 1. The suggested antenna dimensions are determined by optimizing the design with microwave CST studio.

**Figure 3:**
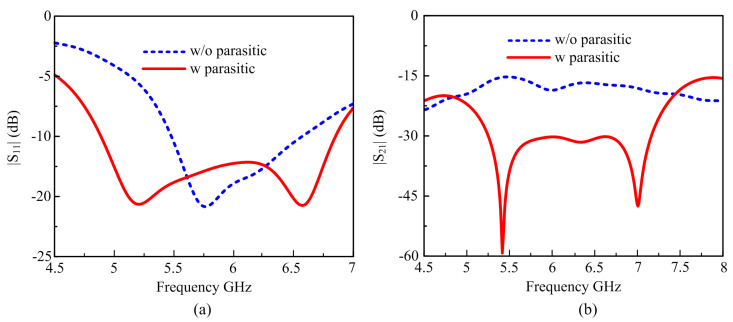
(**a**) Simulted|S_11_| without and with parasitic elements. (**b**) simulated |S_21_| without and with parasitic elements.

In [44], a unique circular ring and parasitic element are suggested to reduce the MC of the antenna investigated. In this paper, the square parasitic elements are used around two-port antennas for the whole performance enhancement of the antenna. This part mostly focuses on improving isolation between the antenna elements and AR. The analyses of the suggested antenna without and with parasitic element results are given in Figure 3. It should be noted that our best results have been obtained by the insertion of square parasitic elements periodically around two radiators to achieve the optimum performance of the MIMO-CP antenna.

The parasitic elements produce a wide bandwidth and additional resonance, resulting in the antenna’s overall impedance BW of −10 dB. The simulated |S_11_| of the suggested antenna without and with parasitic elements is shown in Figure 3a. The antenna without parasitic elements has simulated reflection coefficients of |S_11_| > −10 dB BW (5.4–6.57 GHz). However, the suggested parasitic antenna significantly enhances the antenna’s BW. The design with parasitic elements given a wide bandwidth from 4.89 to 6.85 GHz (33.39%) for |S_11_| was <−10 dB. The effect of the parasitic elements on the MC of the antenna is shown in Figure 3b. The antenna without parasitic elements has poor isolation over the entire bandwidth because |S_21_| is above −20 dB, while after the insertion of the parasitic elements, the mutual coupling has been reduced to −20 dB overall bandwidth, as shown in Figure 3b. The simulations result without parasitic shows that narrow impedance BW, quite narrow axial ratio BW, and poor isolation are always undesirable, and this is what the results of the simulations showed. Mutual coupling (MC) was not significantly reduced by this configuration. As a result, the primary goal of this article was to reduce MC, which is improved by parasitic elements.

**Figure 4:**
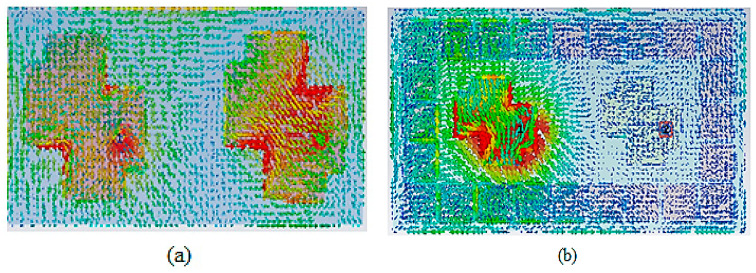
Proposed antenna surface current distribution when Port 1 is excited, and Port 2 is terminated: (**a**) without parasitic elements; (**b**) with parasitic elements.

The suggested MIMO-CP antenna current distribution verifies the impact of the parasitic elements on reducing MC. The surface current at 5.4 GHz with and without parasitic elements is shown in Figure 4. A high MC is obtained between the patches when Port 1 is excited, and Port 2 is terminated with a 50-ohm impedance because the current is strongly coupled to another radiator without parasitic elements, as shown in Figure 4a. Figure 4b shows that with the proposed position of parasitic elements, the current density is reduced, unusually between the two radiating elements. As a result, these analyses assist us in determining the optimal position of the parasitic elements for the lowest MC between the patches. The introduction of the parasitic elements around the patches jammed the current from another side, which enhanced isolation among the two radiators.

**Figure 5:**
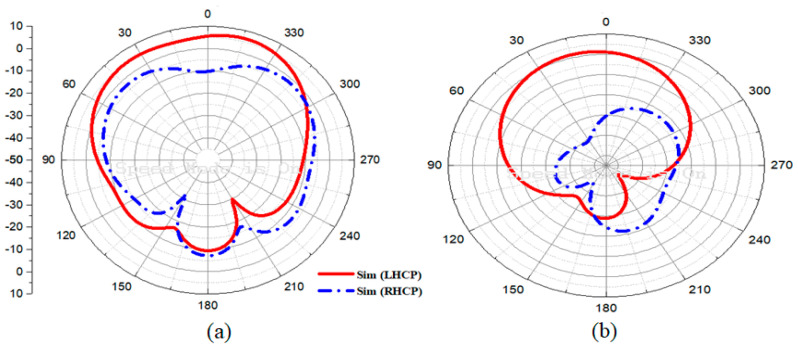
Simulated radiation pattern: (**a**) LHCP and RHCP at 0°; (**b**) LHCP and RHCP at 90°.

**Figure 6:**
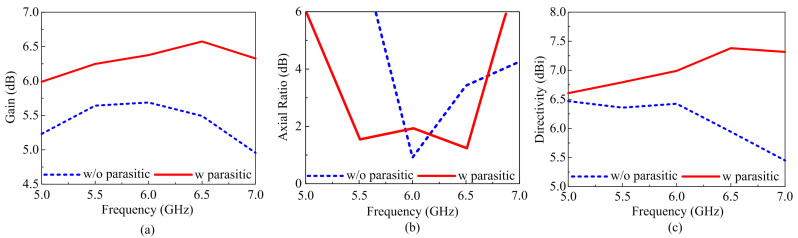
(**a**) Simulated gain of the antenna with and without parasitic. (**b**) Axial ratio of the antenna with and without parasitic. (**c**) Directivity of the antenna with and without parasitic.

Figure 5 shows a far-field radiation pattern at the minimum AR at 5.4 GHz in the φ = 0° and ∅ = 90° planes. When Port 1 is excited, the LHCP field becomes stronger. Figure 5 clearly shows that the LHCP field is stronger in the broadside direction than the RHCP field. The antenna deals with the LHCP sense of polarization because the RHCP is very insignificant compared to the LHCP displaying high polarization isolation. The MIMO-CP antenna gain without and with parasitic elements is shown in Figure 6a. The antenna gain without parasitic elements is seen to be 5.50 dBi; however, the gain with parasitic is enhanced up to 6.45 dBi due to the good efficiency of the antenna acknowledgments to the parasitic elements. The simulated AR of the antenna without and with parasitic elements is shown in Figure 6b. The proposed antenna without a parasitic element has a narrow AR of 5.9 to 6.3 GHz. A wide 3-dB AR BW was achieved by the suggested antenna with a parasitic of 18.25% (5.41–6.58 GHz). Figure 6c shows the directivity of the proposed MIMO-CP antenna with and without parasitic elements. As expected from the parasitic elements, the proposed antenna with parasitic elements offers a slightly higher value of directivity compared to the MIMO antenna without parasitic elements. 

## 4. Proposed Antenna Parametric Study

The parametric studies of the suggested antenna were approved on the CST simulator for the various design parameters, as these parameters show a critical character in controlling the antenna performances. The outcome of square cut at square patch length and width increases or decreases has been studied to attain the best results.

**Figure 7:**
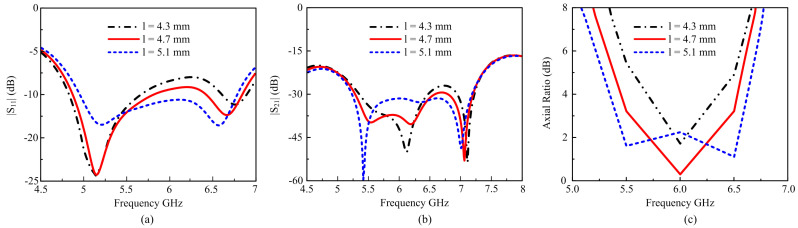
Simulated results for the antennas with different square cuts of l: (**a**) |S_11_|; (**b**) |S_21_| (**c**) Axial ratio.

The suggested antenna parametric is presented using increased and decreased length and width of square cut from the design, and the finest numbers for enhanced performance were selected, as shown in Figure 7. The dimension of cut l varied from 4.3 mm to 5.5 mm. As the dimension decreased, it is clear from Figure 7a that the best performance for |S_11_| is attained when l = 4.3 mm, but the |S_21_| value for the corresponding circumstance increases and has a poor axial ratio. When the value of l increases, the |S_11_| remains almost unaffected and |S_21_| decreases but still has a poor axial ratio. It should be noted that our best results have been simulated numerically to achieve optimum performance for different dimensions of the square cut chosen (5.1 mm), and they have wide bandwidth, wide axial ratio, and reduced mutual coupling within the operating bandwidth.

**Figure 8:**
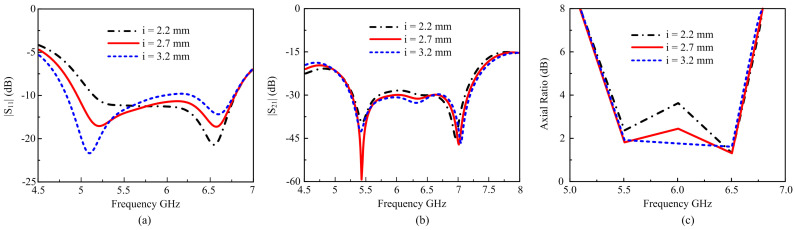
Simulated results for opposite square cut at square patch i: (**a**) |S_11_|; (**b**) |S_21_|; (**c**) Axial ratio.

The suggested antenna is similarly simulated for the opposite corner square cut dimension i at the square patches, as shown in Figure 8. Dimension i of the square cut varies from 2.2 mm to 3.7 mm. When the dimension of i decreases from 2.7 mm, poor |S_11_| return loss is achieved along with poor isolation, and the AR at 2.2 mm. When the dimension increased from 2.7 mm, the |S_11_| and the axial ratio became narrow, but the isolation was not affected further. It is notable from Figure 8 that there was a good wide bandwidth and better isolation, and the axial ratio obtained for gap I = 2.7 mm.

## 5. Experimental Results and Discussion

**Figure 9:**
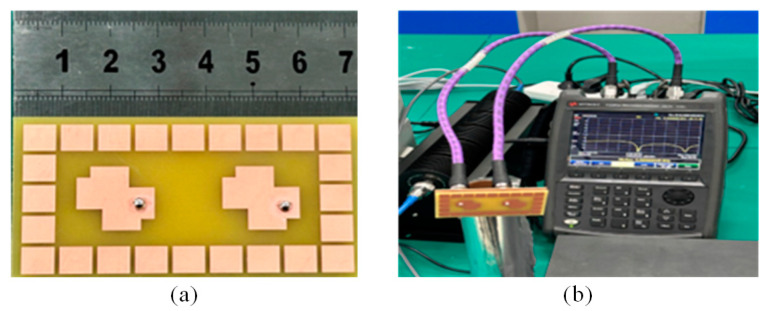
(**a**) Snapshot of the fabricated prototype. (**b**) Photo of measurement setup.

**Figure 10:**
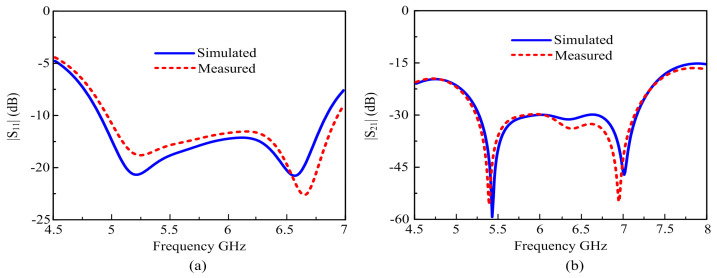
Simulated and measured S-Parameters: (**a)** |S_11_|; (**b)** |S_21_|.

The suggested MIMO antenna, with a parasitic fabricated photograph, is shown in Figure 9. It was measured by a Keysight-made N9916A PNA network analyzer. The S-parameter measurement and simulation results for the suggested antenna were reliable, as shown in Figure 10.

According to the reflection response, the antenna offers an impedance BW (|S_11_|≥ −10 dB) between 4.89 GHz and 6.85 GHz. It can also be noted that the |S_21_| among the two ports was below 20 dB for both the measured and simulated results, as shown in Figure 10b. The outcomes of simulation and measurement are usually similar. The slight discrepancy among the results from measurements and simulation may be caused by fabrication mistakes and the poorly welded or soldered joint of the SMA connector.

**Figure 11:**
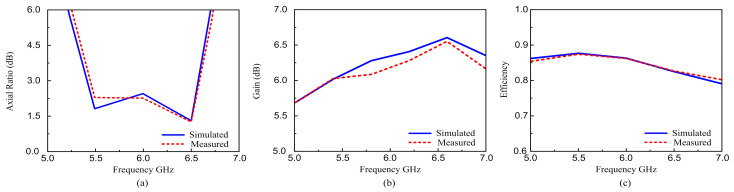
Simulated and measured axial ratio, gain, and total efficiency of the proposed antenna: (**a**) Axial ratio; (**b**) Gain; (**c**) Radiation efficiency.

Figure 11 shows the variation of the AR versus frequency. The simulation results were generally consistent with the measured values, with only a minor difference in bandwidth. Figure 11a presents the measured and simulated gain, AR, and efficiency BW. The simulated 3-dB ARBW is approximately 18.2% (5.40–6.51 GHz), while the measured axial ratio is nearly identical to the simulated one, as shown in Figure 11a. Figure 11b shows that the suggested MIMO CP antenna has a measured peak gain of almost 6.46 dBic and a simulated peak gain of 6.53 dBic. Figure 11c shows that the radiation efficiency is more than 80% across the entire frequency band.

## 6. Diversity Performance of Suggested Antenna

The performance of the MIMO CP antenna is computed using vital parameters that are well-defined to describe MIMO antenna systems, such as the Envelope Correlation Coefficient (ECC), Diversity Gain (DG), CCL, and Total Active Reflection Coefficient (TARC). The diversity presentation of the suggested antenna has been estimated by CST Microwave Studio. The following section provides a detailed illustration of these parameters.

### 6.1. Envelope Correlation Coefficient

The key parameter used to assess the effectiveness of MIMO are the ECC. The suggested antenna diversity is observed in terms of ECC to certify virtuous MIMO performance. ECC characterizes the correlation between signals received by the antenna. Embedded 3D far-field radiation patterns or scattering characteristics can be used to calculate ECC. Studying any lossy antenna, it is important to remember that estimating ECC values for S-parameters is ineffective and drastically underestimates its standards. The radiation efficiency and S-parameters can be used to analyze it for a two-port MIMO antenna, as presented in Equation (1) [45].

(1) $$\mathrm{ECC}=\frac{\left|S_{11}\times S_{12}+S_{21}\times S_{22}\right|}{\left(1-|S_{11}{|}^{2}-|S_{12}{|}^{2}(1-|S_{21}{|}^{2}-|S_{22}{|}^{2}\right)}$$

**Figure 12:**
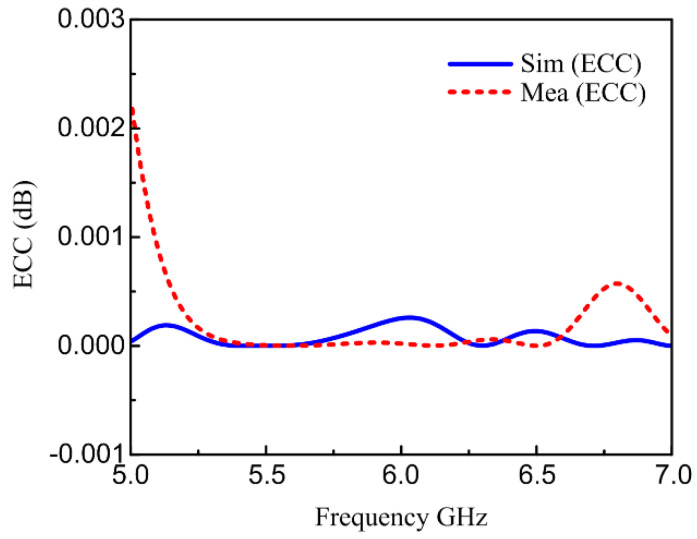
Proposed measured and simulated ECC.

The suggested two-element antenna ECC for measured and simulated radiation fields is presented in Figure 12. The ECC value must be less than 0.5 for better MIMO systems [46]. The measured and simulated ECC are shown in Figure 12. As presented in the figure, the measured and simulated ECC values are below 0.002 and 0.001, respectively, in the frequency band. The values of the simulated and measured ECC are well below the suitable limit of 0.5. Therefore, a good MIMO performance of the proposed antenna is guaranteed.

### 6.2. Diversity Gain

Diversity gain is the second major parameter of the MIMO system. The characteristics of diversity are frequently attained when transmitters receive different transmission stream formats via different channel pathways. It was observed that the DG value was close to 10, signifying better diversity performance. The DG value can be calculated by ECC in Equation (2) [47].

(2) $$\mathrm{DG}=10\times \sqrt{1-{\left(\mathrm{ECC}\right)}^{2}}$$

**Figure 13:**
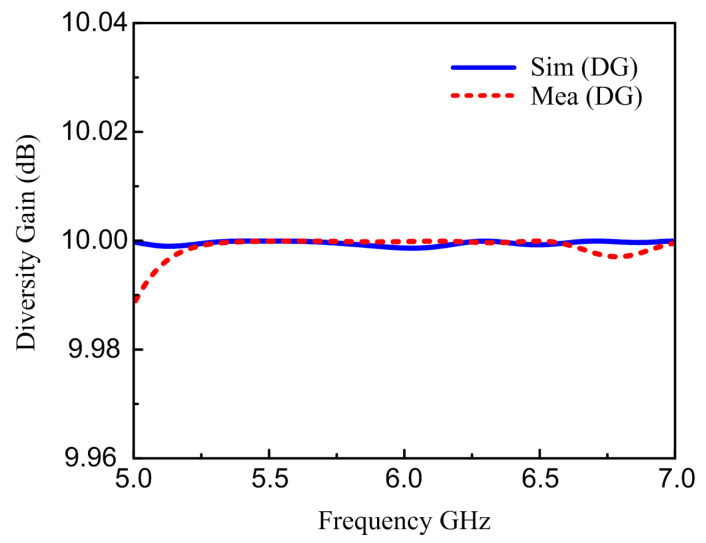
Simulated and measured diversity gain.

Figure 13 depicts the simulated and measured DG of the suggested antennas, which is close to 10 dB.

### 6.3. Channel Capacity Loss

**Figure 14:**
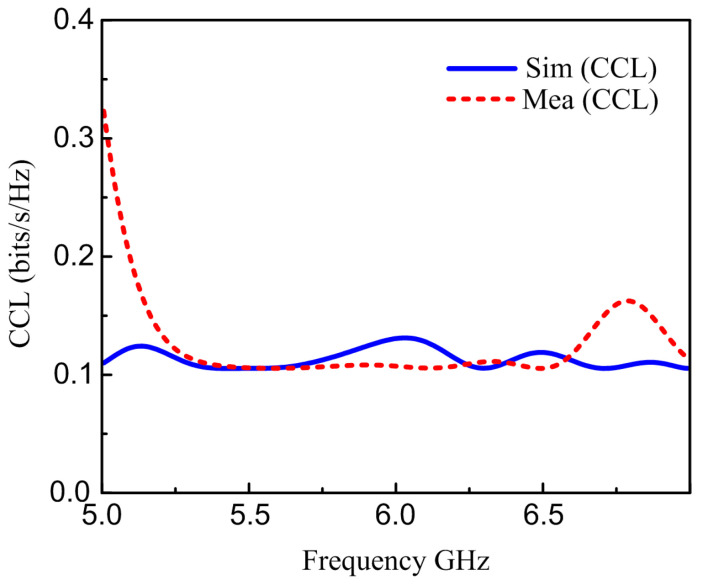
Simulated and measured CCL.

CCL is the next key factor used to calculate diversity presentation. The term CCL stands for the highest permitted message broadcast rate at which signals can be sent continuously over the communication system. The CCL should not go over 0.4 bits/s/Hz for reliable communication. The suggested antenna CCL is calculated by using S-parameters [48]. Figure 14 shows the CCL calculated from the S-Parameters. The CCL values of the designed antenna attain less than 0.4 bits/s/Hz.

(3) $$C_{Loss}=-\log_{2}\det(\Psi^{R})$$

where $\psi^{R}$ is the correlated matrix of the receiver and is defined by

(4) $$\psi^{R}=\left[\begin{array}{cc} \psi_{ii} & \psi_{ij}\\ \psi_{ji} & \psi_{jj} \end{array}\right]$$

(5) $$\psi_{ii}=1-\left(\left|s_{ii}{|}^{2}-\right|s_{ij}{|}^{2}\right)\;$$

(6) $$\psi_{jj}=1-\left(\left|s_{ij}{|}^{2}-\right|s_{jj}{|}^{2}\right)$$

(7) $$\psi_{ij}=-\left(s_{ii}^{*}s_{ij}+s_{ji}^{*}s_{jj}\right)$$

(8) $$\psi_{ji}=-\left(s_{jj}^{*}s_{ji}+s_{ij}^{*}s_{ii}\right)$$

### 6.4. Total Active Reflection Coefficient

**Figure 15:**
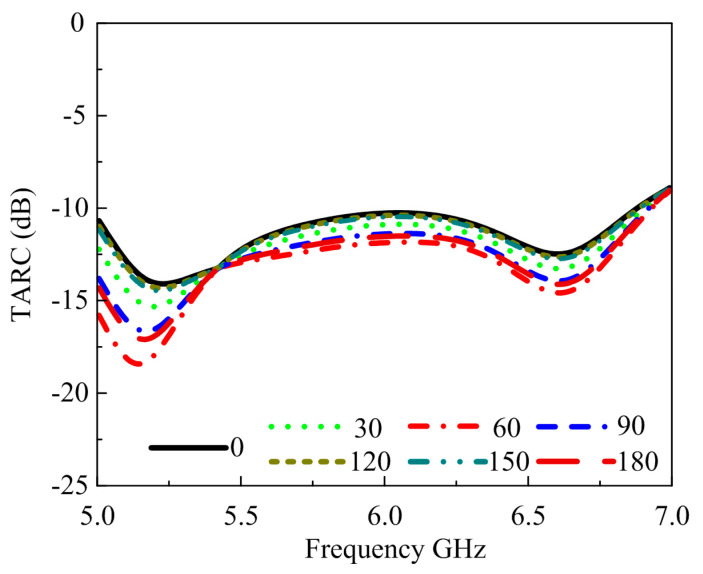
Proposed simulated TARC.

Calculating the scattering matrix remains insufficient for estimating the radiation presentation of an MIMO system. For the MIMO systems, the TARC is well-defined as the ratio of reflected incident power [49]. TARC is represented by scattering parameters in a two-element MIMO antenna, as shown below:

(9) $$\Gamma_{a}^{t}=\frac{\sqrt{\sum_{j}^{m}|bj{|}^{2}}}{\sqrt{\sum_{j}^{m}|aj{|}^{2}}}$$

where *bj* represents the reflected wave and *aj* denotes the incident wave; TARC dependence on S-parameter is defined as [50].

(10) $$\frac{TARC=\sqrt{(|s_{11}+s_{12}e^{j\phi }{|}^{2}+(|s_{21}+s_{22}e^{j\phi }{|}^{2}}}{2}$$

where *ϕ* is the range of the random phase of the return signal between 0 and π; it has a Gaussian distribution owing to the multipath propagation channel. The TARC ranges from 0 to 1, where TARC = 0 refers to the situation when all power coming in is radiated and none is reflected, and TARC = 1 refers to the situation where total the power coming in is reflected and nothing is radiated. Figure 15 depicts the TARC curves for various input phase values, ranging from 0 to π, with periods of 30°. These curves indicate that the design samples maintain less than −10 dB TARC over the whole proposed bandwidth for various input phases.

## 7. Comparison Performance with Previous Work

To explain the novelty of the proposed MIMO-CP antenna, Table 2 is presented. Table 2 compares the presentation of the designed MIMO antenna to recent related work published in the literature. The comparison is based on the antenna dimensions, operating bandwidth, peak isolation, and peak gain. The suggested antenna also offers high-isolation between the antenna radiators as well as wide bandwidth, high gain, and circular polarization. Table 3 compares the suggested MIMO-CP antenna with other current works in the literature that feature comparable MIMO-CP designs with significant isolation. Due to some performance metrics, the comparison is made with overall antenna size, operating bandwidth, peak isolation, and mutual coupling reduction techniques. The gain of the proposed antenna was higher or comparable with that of other recent works on antennas. The CP bandwidth was also comparatively wider in the case of a two-port MIMO antenna.

The antenna of [34,39] is small compared to our proposed antenna and has maximum isolation, but these designs suffer from low gain, narrow bandwidth, and axial ratio. Furthermore, the antenna in [17] gives better peak gains, while its size is large and suffers from narrow bandwidth and AR. Our proposed design uses a novel technique for better bandwidth, a wide axial ratio, and high peak gain with better isolation capability. Our proposed antenna’s main aim is to reduce MC. The suggested MIMO antenna provides better isolation with a compact size, making it a potential candidate for MIMO system applications.

## 8. Conclusions

In this article, a wide-band MIMO CP antenna is designed for WLAN application. The proposed antenna has a simple configuration with a width and length of 40 × 70 mm^2^. The square patch is cut at all corners to attain CP by using the concept of circular polarization. The design Improved impedance matching, gain, mutual coupling, and AR, which can all be attained by placing parasitic elements periodically around the two antennas. The antenna impedance BW ranges from 4.89 GHz to 6.85 GHz and AR ≤ 3dB from 5.41 to 6.58 GHz; peak isolation |S_21_| ≤ −60 dB within the |S_11_| frequency band. At the resonant frequency, the radiation efficiency and peak gain are 6.5 dB and 84%, respectively. The envelope correlation coefficient is 0.001 and the diversity gain is almost 10 dB. The measured and simulated results from the proposed antenna are appropriate for WLAN applications.

## Figures and Tables

**Table 1:** Dimensions of the suggested antenna.

Parameters	Size (mm)	Parameters	Size (mm)	Parameters	Size (mm)
W_F_	70	L_F_	40	s	13.5
l h	5.1 1.6	W_i_ w_g_	7.6 6.4	i g	2.7 1

**Table 2:** Performance of proposed MIMO work compared with previously published work.

References	Size (mm)	Isolation (dB)	Bandwidth (GHz)	Gain (dBi)	MC Techniques
[12]	139.3 × 44	40	5.50−5.68	Not given	Complementary of the split ring resonator
[21]	110 × 110	35	5.65−6.10	6.2	Metasurface
[22]	32 × 60	24	5.68−6.05	7.98	Metamaterial
[23]	70 × 60	41	5.70−6.20	9.4	DNG Metameterial Superstrate
[24]	100 × 50	25	5.10−6.0	Not given	DGS
[26]	68 × 40	45	5.71−6.10	Not given	EBG
[27]	57 × 32	25	5.50−5.80	6.4	Parallel couples Resonator
[28]	47 × 32	45	3.0−7.70	3	Comb Shaped
[29]	35 × 33	30	3.10−5.0	3.2	Neutralization Line
[30]	95.9 × 38.2	24	2.43−2.50	4.68	Fractal EBG
Proposed work	70 × 40	64	4.89−6.85	6.45	Square Parasitic Elements

**Table 3:** Comparison with another MIMO antenna based on CP.

References	Size (mm)	Bandwidth (GHz)	ARBW CP	Isolation (dB)	Gain (dB)	Isolation Techniques
[17]	96 × 96	2.36–2.53	2.30–2.50	25	8.0	slot
[34]	60 × 33	3.9–4.2	3.97–4.30	37.5	3.4	Annular ring patch with stubs
[36]	65 × 45	5.1–5.35	4.90–5.40	25	4	T-shaped slot and Endfire antenna
[37]	100 × 150	2.47–2.55	2.55–2.60	20	6.1	Hybrid Techniques
[38]	27.69 × 97	5.49–6.02	5.77–5.86	33	5.34	Slot Techniques
[39]	40 × 50	5.50–5.80	5.55–5.60	25	4.70	DGS
[40]	40 × 65	5.20–6.08	5.20–5.58	20	4.01	Dielectric Resonator
[41]	80 × 80	5.71–8.20	5.77–8.08	15	3.8	Dielectric Resonator
[43]	66 × 66	1.8–2.6	5.2–5.58	25	4.0	Parasitic Line Patch
Proposed Work	70 × 40	4.89–6.85	5.41–6.57	64	6.45	Square Parasitic Elements

## Data Availability

The data that support the findings of this study are available from the corresponding author upon reasonable request.
